# Comparison of hemodynamic effects of chest compression delivered via machine or human in asphyxiated piglets

**DOI:** 10.1038/s41390-023-02827-4

**Published:** 2023-09-23

**Authors:** Megan O’Reilly, Tze-Fun Lee, Po-Yin Cheung, Georg M. Schmölzer

**Affiliations:** 1 Centre for the Studies of Asphyxia and Resuscitation, Neonatal Research Unit, Royal Alexandra Hospital, Edmonton, AB Canada; 2 Department of Pediatrics, University of Alberta, Edmonton, AB Canada

## Abstract

**Background:**

High-quality chest compressions (CC) are an important factor of neonatal resuscitation. Mechanical CC devices may provide superior CC delivery and improve resuscitation outcomes. We aimed to compare the hemodynamic effects of CC delivered by machine and human using a neonatal piglet model.

**Methods:**

Twelve asphyxiated piglets were randomized to receive CC during resuscitation using an automated mechanical CC device (“machine”) or the two-thumb encircling technique (“human”). CC was superimposed with sustained inflations.

**Results:**

Twelve newborn piglets (age 0–3 days, weight 2.12 ± 0.17 kg) were included in the study. Machine-delivered CC resulted in an increase in stroke volume, and minimum and maximum rate of left ventricle pressure change (dp/dt_min_ and dp/dt_max_) compared to human-delivered CC.

**Conclusions:**

During machine-delivered CC, stroke volume and left ventricular contractility were significantly improved. Mechanical CC devices may provide improved cardiopulmonary resuscitation outcomes in neonatal cardiac arrest induced by asphyxia.

**Impact:**

- Machine chest compression leads to changes in hemodynamic parameters during resuscitation of asphyxiated neonatal piglets, namely greater stroke volume and left ventricular contractility, compared with standard two-thumb compression technique. Mechanical chest compression devices may provide improved cardiopulmonary resuscitation outcomes in neonatal and pediatric asphyxia-induced cardiac arrest.

## Introduction

The incidence of cardiopulmonary resuscitation (CPR) in the delivery room is 0.1% of term and up to 15% of preterm infants) with high incidence of morbidities and mortality.^1^ This raises the question as to whether improved CPR techniques could improve outcomes. While high-quality chest compressions (CC) are an important part of successful CPR,^2^ rescuer fatigue is an important concern, as this might decrease effectiveness of chest compression (CC). During simulated neonatal CPR, a decrease in CC performance quality and fatigue was observed as early as 3.5 min after beginning CC.^3^

Alternatively, mechanical CC devices might be used as this could theoretically reduce problems associated with fatigue, manpower, and CPR consistency. However, clinical trials in adults have had conflicting results reporting either improved or reduced survival or faster or slower time to return of spontaneous circulation.^4^ A recent meta-analysis included 15 studies (18,474 patients) reported no difference in time to return of spontaneous circulation in out-of-hospital adult cardiac arrest [Odds Ratio (95% Confidence Interval) 1.16, 0.97–1.39, *p* = 0.11] between mechanical and manual compressions.^4^

We developed a mechanical CC machine,^5–8^ which allows for systematic examination of machine-delivered CC compared to human-delivered CC. We aimed to compare the hemodynamic effects of machine versus human CC during CPR in our neonatal piglet model of cardiac arrest induced by asphyxia.

## Methods

**Fig. 1:**
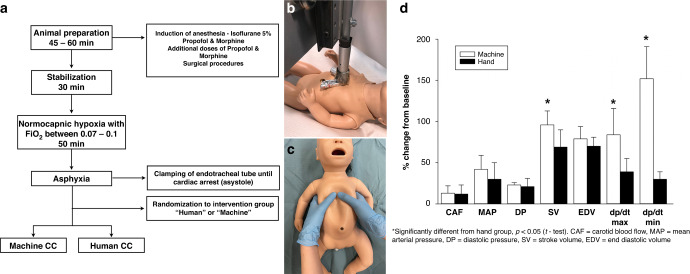
Study design and hemodynamic outcomes during chest compressions. **a** Study flow diagram: CC chest compression, FiO_2_ fraction of inspired oxygen. **b** Machine chest compressions: shape and placement of the compression point on a manikin. **c** Human chest compressions: placement of the hands on a manikin for the 2-thumb encircling technique. **d** Hemodynamic outcomes: *significantly different from human group (*p* < 0.05); CAF carotid artery flow, MAP mean arterial blood pressure, DP diastolic blood pressure, SV stroke volume, EDV end-diastolic volume, dp/dt_max_ and dp/dt_min_, maximum and minimum rate of left ventricular pressure change.

Term newborn mixed breed post-transitional piglets were obtained on the day of experimentation from the University Swine Research Technology Center. All experiments were conducted in accordance with the guidelines and approval of the Animal Care and Use Committee (Health Sciences), University of Alberta (AUP00002844), presented according to the ARRIVE guidelines^9^ (supplement), and registered at preclinicaltrials.eu (PCTE0000365). A graphical display of the study protocol is presented in Fig. 1a.

### Animal preparation

Piglets were instrumented as previously described with some modifications.^10,11^ Following the induction of anesthesia using isoflurane, piglets were tracheotomized and mechanically ventilated (Acutronic, Hirzel, Switzerland) with a respiratory rate of 16–20/min and pressure of 20/5 cmH_2_O. Oxygen saturation was kept within 90–100%. A 5-French Argyle® (Klein-Baker Medical Inc. San Antonio, TX) double-lumen catheter was inserted via the right femoral vein for administration of fluids and medications. The glucose level and hydration were maintained with an intravenous infusion of 5% dextrose at 10 mL/kg/h. During the experiment anesthesia was maintained with intravenous propofol 5–10 mg/kg/h and morphine 0.1 mg/kg/h. After surgery, the piglets were stabilized for 1 h.

### Hemodynamic parameters

The right common carotid artery was also exposed and encircled with a real-time ultrasonic flow probe (2 mm; Transonic Systems Inc., Ithica, NY) to measure carotid blood flow. A 5-French Argyle® single-lumen catheter was inserted above the right renal artery via the femoral artery for continuous arterial blood pressure monitoring using a Hewlett Packard 78833B monitor (Hewlett Packard Co., Palo Alto, CA), in addition to arterial blood gas measurements. A Millar catheter (MPVS Ultra, ADInstruments, Houston, TX) was inserted into the left ventricle (LV) via the left common carotid artery for continuous measurement of stroke volume, end-diastolic volume, and left ventricular contractile function (dp/dt_max_, dp/dt_min_).

### Mechanical chest compression (CC) machine

We used a custom-designed mechanical CC machine for mechanical CC delivery (machine group only).^5–8^ The CC machine delivers various CC rates (50–200 per minute), anterior-posterior (AP) chest compression depths (10–70%), acceleration of compressions (100–1000 cm/s^2^), recoil speed (1–100 cm/s), steps per revolution (400–1200 steps per revolution), and varying duty cycle.

### Depth measurement

The depth was measured using an infrared transmitter and receiver (Gikfun, Guangdong, China) with the transmitter located on the chest and the receiver stationary on the machine. The depth was recorded with the Arduino Software (Somervile, MA) with a sample rate of 200 Hz.

### Experimental protocol

Following surgical instrumentation and stabilization, piglets were exposed to 50 min of normocapnic hypoxia. Following hypoxia, piglets were randomized to group allocation by opening a subsequently numbered, sealed brown envelope containing the assignment “machine” or “human” (Fig. 1a), and then were exposed to asphyxia. Piglets randomized to “machine” group were placed onto the automated CC machine, which was placed onto the surgical bed, before commencing asphyxia. Asphyxia was achieved by disconnecting the ventilator and clamping the endotracheal tube until asystole. Asystole was defined as no heart rate audible during auscultation and zero blood flow in the carotid artery. One minute after cardiac arrest (asystole), CC was initiated, using 21% oxygen, with an AP chest diameter depth of 33%, continuous CC during sustained inflation with a peak inflation pressure of 30 cmH_2_O for 30 s. The sustained inflation was interrupted for 1 s before a further 30 s of sustained inflation was provided. Piglets randomized to “machine” group received mechanically performed CC using the automated CC machine, with an acceleration of compression of 500 cm/s^2^, recoil speed of 50 cm/s, and CC rate of 90/min. Piglets randomized to “human” group received CC using the two-thumb encircling technique,^8^ with a CC a rate of 90/min using a metronome, performed by a single operator (GMS) in all piglets. This was a non-surviving animal model with no return of spontaneous circulation, as the purpose of the study was to examine the hemodynamic effect of machine versus human CC.

### Data collection and analysis

Demographics of study piglets were recorded. Transonic flow probes and pressure transducer outputs were digitized and recorded with LabChart® programming software (ADInstruments, Houston, TX). CC were performed for 60 s for each group in each piglet. Ten-second hemodynamic recordings immediately before hypoxia (baseline) and in the middle of CC (30–40 s after starting CC) were used as comparison. The data are presented as mean ± standard deviation (SD) for normally distributed continuous variables. The data was tested for normality (Shapiro–Wilk and Kolmogorov–Smirnov test) and compared using Student’s *t*-test. *P*-values are 2-sided and *p* < 0.05 was considered statistically significant. Statistical analyses were performed with SigmaPlot (Systat Software Inc, San Jose).

## Results

Twelve newborn mixed breed piglets (0–3 days of age, weighing 2.12 ± 0.17 kg) were included. Data for hemodynamic parameters are presented in Fig. 1d and Table 1. Blood gas parameters are presented in Table 1. There was no return of spontaneous circulation during CC in all piglets. The anterior posterior depth with machine-delivered CC was 32 ± 2% and with human-delivered CC was 38 ± 2%.

**Table 1:** Blood gas and hemodynamic parameters.

	Machine (*n* = 6)	Human (*n* = 6)	*p*-value
Baseline			
Arterial pH	7.48 (0.03)	7.47 (0.05)	0.68
PaCO_2_ (torr)	35.8 (3.5)	34.8 (3.4)	0.63
Base excess (mmol/L)	3.0 (3.0)	1.7 (1.4)	0.36
Lactate (mmol/L)	3.4 (0.8)	3.6 (0.9)	0.69
Carotid blood flow (mL/min)	81 (22)	69 (26)	0.41
Mean arterial blood pressure (mmHg)	67 (9)	55 (13)	0.09
Diastolic blood pressure (mmHg)	53 (8)	43 (10)	0.08
Stroke volume (mL/kg)	1.5 (0.6)	1.4 (0.3)	0.72
End diastolic volume (mL/kg)	4.9 (1.4)	3.7 (1.6)	0.20
dp/dt_max_ (mmHg)	2823 (590)	2801 (550)	0.95
dp/dt_min_ (mmHg)	−3552 (870)	−3401 (1163)	0.80
At end of asphyxia			
Arterial pH	6.61 (0.10)	6.82 (0.07)	0.001
PaCO_2_ (torr)	93.5 (27.3)	80.5 (15.9)	0.34
Base excess (mmol/L)	−27.9 (2.9)	−21.8 (2.6)	0.003
Lactate (mmol/L)	16.3 (2.5)	15.4 (0.8)	0.42
During chest compressions			
Carotid blood flow (mL/min)	10 (7)	11 (6)	0.79
Mean arterial blood pressure (mmHg)	26 (12)	15 (10)	0.11
Diastolic blood pressure (mmHg)	12 (2)	8 (4)	0.05
Stroke volume (mL/kg)	1.4 (0.3)	0.9 (0.3)	0.02
End diastolic volume (mL/kg)	3.9 (1.3)	2.6 (1.4)	0.13
dp/dt_max_ (mmHg)	2324 (854)	1096 (434)	0.01
dp/dt_min_ (mmHg)	−5389 (2036)	−1002 (377)	<0.001

Data are presented as mean (SD).

There were no differences in the baseline hemodynamic parameters (Table 1) or baseline blood gas parameters between the groups (Table 1). During CC stroke volume was significantly higher in piglets receiving machine CC compared to human CC (Fig. 1d and Table 1). Similarly, the dp/dt_max_ and dp/dt_min_ were significantly different in the machine CC group compared to human CC (Fig. 1d and Table 1), indicating improved left ventricular contractile function. Of note, arterial pH and base excess at the end of asphyxia (Table 1) was significantly lower in the machine group compared to the human group, indicating a worse metabolic acidosis. Nevertheless, piglets in the machine group presented with improved hemodynamic outcomes during CC compared to piglets receiving human CC.

## Discussion

Evidence on the use of mechanical CC devices for neonatal CPR is lacking. Adult animal studies as well as adult clinical trials reported significantly improved hemodynamics during mechanical CC including systolic and diastolic arterial blood pressure, right atrial pressure, and coronary perfusion pressure.^4,12^ However, when all available randomized trials were combined in a meta-analysis reported no difference in time to return of spontaneous circulation after out-of-hospital adult cardiac arrest between mechanical and manual compressions.

In the current study, we showed that during CPR in asphyxiated neonatal piglets machine-delivered CC improved hemodynamic outcomes compared to human-delivered CC using the two-thumb encircling technique. Machine-delivered CC resulted in a significantly improved stroke volume and left ventricular contractility. Furthermore, diastolic blood pressure, an important factor for myocardial perfusion, was of borderline significance with 12 ± 2 mmHg with machine-delivered CC compared to 8 ± 4 mmHg with human-delivered CC (*p* = 0.05) (Table 1).

We aimed to deliver a depth of 33% of the anterior posterior chest diameter with both approaches, however, with the machine-delivered CC, it was ~32% and 38% with the human-delivered CC. Currently, there are no feedback mechanism to assure optimal CC depth during real-life CPR and it might be possible that healthcare providers compress the chest deeper than the recommended 33%. Bruckner et al. reported that CC depth influenced hemodynamic parameters and that carotid blood flow and systolic blood pressure were the highest using a CC depth of 40% anterior posterior chest diameter.^5^ Interestingly, despite higher CC depth during human-delivered CC, the stroke volume and the left ventricular function were significantly lower than those of machine-delivered CC. These results challenge the role of CC depth in CPR and lead us to speculate that the force used to compress the chest contributes significantly to the stroke volume and left ventricular function during CC.

It is probable that an improved stroke volume combined with superior ventricular contractility led to improved cardiac output and might increase the possibility of return of spontaneous circulation. Notably, piglets in the machine group presented with more severe metabolic acidosis yet experienced superior hemodynamic outcomes compared to piglets in the human CC group. This indicates that our automated CC machine can deliver consistent high-quality CC that directly impacts hemodynamic parameters.

In the current study,^11^ we used our novel CPR technique, which combines continuous CC with sustained inflation ventilation (=CC + SI). CC + SI is mentioned in the knowledge gap section of the Neonatal Consensus of Science and Treatment Recommendations,^1^ but it is currently not the recommended practice. Using the currently recommended technique of 3:1 compression-to-ventilation ratio may have yielded different outcomes. However, our mechanical CC devices can only provide continuous CC.

## Limitations

Our use of a piglet asphyxia model is a great strength of this translational study, as this model closely simulates delivery room events, with the gradual onset of severe asphyxia leading to asystole. Our asphyxia model uses piglets that have already undergone the fetal-to-neonatal transition, were sedated/anesthetized, and uses tracheostomy with a tightly sealed endotracheal tube to prevent leak; which does not occur in the delivery room, which are limitation of our model.

## Conclusion

Mechanical chest compression using an automated device was associated with changes in hemodynamic parameters during cardiopulmonary resuscitation in a neonatal animal model. Machine-delivered chest compressions resulted in greater stroke volume and left ventricular contractility. Mechanical chest compression devices may provide improved cardiopulmonary resuscitation outcomes in neonatal and pediatric cardiac arrest induced by asphyxia.

### Supplementary information

Supplementary information

## Data Availability

All data generated or analyzed during this study are included in this published article. Data used to generate the results reported in this study will be made available following publication to researchers who provide a methodologically sound proposal.

## References

[1] Wyckoff MH (2020). Neonatal life support: 2020 international consensus on cardiopulmonary resuscitation and emergency cardiovascular care science with treatment recommendations. Circulation.

[2] Solevåg AL, Cheung P-Y, O’Reilly M, Schmölzer GM (2016). A review of approaches to optimise chest compressions in the resuscitation of asphyxiated newborns. Arch. Dis. Child Fetal Neonatal Ed..

[3] Enriquez D, Meritano J, Shah B, Song C, Szyld E (2018). Fatigue during chest compression using a neonatal patient simulator. Am. J. Perinat..

[4] Sheraton M, Columbus J, Surani S, Chopra R, Kashyap R (2021). Effectiveness of mechanical chest compression devices over manual cardiopulmonary resuscitation: a systematic review with meta-analysis and trial sequential analysis. West J. Emerg. Med..

[5] Bruckner M (2021). Effects of varying chest compression depths on carotid blood flow and blood pressure in asphyxiated piglets. Arch. Dis. Child Fetal Neonatal Ed..

[6] Bruckner M (2022). Assessment of optimal chest compression depth during neonatal cardiopulmonary resuscitation: a randomised controlled animal trial. Arch. Dis. Child Fetal Neonatal Ed..

[7] Bruckner M (2023). Haemodynamic changes with varying chest compression rates in asphyxiated piglets. Arch. Dis. Child Fetal Neonatal Ed..

[8] Bruckner M (2022). Chest compression rates of 90/min versus 180/min during neonatal cardiopulmonary resuscitation: a randomized controlled animal trial. Children.

[9] du Sert NP (2020). *The ARRIVE guidelines 2.0: Updated guidelines for repor*ting animal research. Exp. Physiol..

[10] Cheung, P.-Y., Gill, R. S. & Bigam D. L. A swine model of neonatal asphyxia. Journal of visualized experiments: JoVE. **56**, 3166 (2011). 10.3791/3166 PMC3227176 22006174

[11] Schmölzer GM (2013). Cardiopulmonary resuscitation with chest compressions during sustained inflations: a new technique of neonatal resuscitation that improves recovery and survival in a neonatal porcine model. Circulation.

[12] Magliocca A (2019). LUCAS versus manual chest compression during ambulance transport: a hemodynamic study in a porcine model of cardiac arrest. J. Am. Hear Assoc. Cardiovasc Cerebrovasc. Dis..

